# Positive feedback loop between mitochondrial fission and Notch signaling promotes survivin-mediated survival of TNBC cells

**DOI:** 10.1038/s41419-018-1083-y

**Published:** 2018-10-15

**Authors:** Li Chen, Jing Zhang, Zhuomin Lyu, Yibing Chen, Xiaoying Ji, Haiyan Cao, Mingpeng Jin, Jianjun Zhu, Jin Yang, Rui Ling, Jinliang Xing, Tingting Ren, Yonggang Lyu

**Affiliations:** 1 State Key Laboratory of Cancer Biology and Experimental Teaching Center of Basic Medicine, Fourth Military Medical University, 710032 Xi’an, China; 2 Department of Pain Treatment, Tangdu Hospital, Fourth Military Medical University, 710038 Xi’an, China; 3 Center of Genetic & Prenatal Diagnosis, First Affiliated Hospital, Zhengzhou University, 450052 Zhengzhou, China; 4 Institute of Preventive Genomic Medicine, School of Life Sciences, Northwest University, 710069 Xi’an, China; 5 Thyroid and Breast Department, Xi’an No. 3 Hospital, 710082 Xi’an, China; 6 Department of Thyroid, Breast and Vascular Surgery, Xijing Hospital, Fourth Military Medical University, 710032 Xi’an, China

## Abstract

Mitochondrial morphology is remodeled by continuous dynamic cycles of fission and fusion. Emerging data have shown that the disturbance of balance between mitochondrial fission and fusion is involved in the progression of several types of neoplasms. However, the status of mitochondrial dynamics and its potential biological roles in breast cancer (BC), particularly in triple negative BC (TNBC) are not fully clear. Here, we reported that the mitochondrial fission was significantly increased in BC tissues, especially in the TNBC tissues, when compared with that in the corresponding peritumor tissues. Meanwhile, our data showed that Drp1 was upregulated, while Mfn1 was downregulated in TNBC. Moreover, elevated mitochondrial fission was associated with poorer prognosis in TNBC patients. Mitochondrial fission promoted the survival of TNBC cells both in vitro and in vivo. Furthermore, we identified a positive feedback loop between mitochondrial fission and Notch signaling pathway in TNBC cells, as proved by the experimental evidence that the activation of Notch signaling enhanced Drp1-mediated mitochondrial fission and Drp1-mediated mitochondrial fission in turn promoted the activation of Notch signaling, which ultimately promoted the cell survival of TNBC via increasing survivin expression level. Inhibition of either Notch1 or Drp1 significantly impaired the activation of the other, leading to the suppression of TNBC cell survival and proliferation. Collectively, our data reveal a novel mechanism that the positive feedback loop between mitochondrial fission and Notch signaling promotes the survival, proliferation and apoptotic resistance of TNBC cells via increasing survivin expression and thus favors cancer progression.

## Introduction

Breast cancer is one of the most common cancer that affects women’s health worldwide^1,2^. Triple negative breast cancer (TNBC) is a subgroup typically characterized by the absence of estrogen receptor (ER), progesterone receptor (PR), and human epidermal growth factor receptor 2 (HER2) expression. Among breast cancer, TNBC is the most difficult to treat, due to its highly aggressive phenotype, low responsiveness to chemotherapeutic reagents, high rate of recurrence, and poor prognosis^3,4^. Therefore, there is an urgent medical need to identify therapeutic targets and develop more effective treatment strategies for TNBC. Encouragingly, emerging data have highlighted some promising molecular therapeutic targets for TNBC, including EGFR, PARP1, mTOR, TGF-β, Notch signaling, Wnt/β-catenin and Hedgehog pathways^3,5^. However, the detailed molecular mechanisms by which these pathways affect the TNBC development and progression remain unclear.

Notch signaling pathway is an evolutionarily conserved signaling pathway that regulates stem cell maintenance, cell fate specification, differentiation, proliferation, motility and survival^3,5,6^. In mammals, the Notch signaling pathway consists of five ligands (Delta-like proteins 1/3/4, Jagged 1/2) and four receptors (Notch1/2/3/4). After the binding of Notch receptors and ligands, Notch is cleaved by a class of enzymes, resulting in the release of active NICD, which is an initiation of notch downstream signaling^7^. Numerous studies have demonstrated that Notch signaling pathway is frequently activated in many types of malignancies and confers a survival advantage on cancer cells, leading to poor clinical outcomes in patients^8–12^. In invasive breast cancer, the elevated expression of Notch signaling members, including Notch receptors and ligands and target molecules has been reported. In addition, it has been reported that Notch1 mRNA expression is significantly increased in basal-like TNBC and strongly correlated with poor survival of patients^13^. Moreover, specific inhibition of Notch1 signaling has a remarkable inhibitory effect on cancer stem cells and thus increases the sensitivity of TNBC to chemotherapeutic reagents^14^.

Many Notch target molecules have been identified, some of which are particularly important in tumorigenesis, including MYC, IGF1-R, and snail homolog 2 (SLUG)^15–17^. Survivin, a unique member of the IAP protein family, serves as a dual regulator of cell division and apoptosis^18^. Mounting evidence has suggested survivin as a pivotal oncoprotein with multiple roles in the regulation of mitosis, suppression of cell death, and enhanced adaptation to cellular stress^19^. Other evidence also suggests that survivin may be a critical molecule in breast cancer, which links to aggressive disease, resistance to apoptosis, and the modulation of HER2 signaling^20^. Survivin expression is regulated by several oncogenic pathways, such as Wnt/β-catenin signaling^19^. Importantly, coexpression of Notch1 and survivin has been found in basal breast cancer^21^. Stimulation of Notch1 increases the survivin expression in TNBC cells, whereas inhibition of Notch reduces the survivin level, suggesting that survivin is a target of Notch in TNBC. However, to date, the pathophysiological roles of Notch-survivin axis in breast cancer progression remain elusive and need to be further assessed.

Mitochondria are highly dynamic and undergo constant fusion and fission, which is essential for maintaining physiological functions of cells. The main mitochondrial fission and fusion proteins are members of the Dynamin family. Notably, dynamin-related protein 1 (Drp1) and mitochondrial fission 1 (FIS1) are essential for mitochondrial fission, whereas Mfn1 (mitofusin 1), Mfn2 and OPA1 (optic atrophy 1 [autosomal dominant]) are required for mitochondrial fusion^22,23^. Recently, cumulative evidence is beginning to reveal the close links between cancer development and unbalanced mitochondrial dynamics^24,25^. A number of studies have shown that disruption of the mitochondrial network exhibits a considerable effect on the apoptosis, autophagy, and invasion of cancer cells^26–28^. More importantly, several studies have indicated the crosstalk between mitochondrial dynamics and Notch signaling^29–32^. Kasahara et al. have shown that mitochondrial fusion directs cardiomyocyte differentiation via calcineurin and Notch signaling^31^. Pal et al. have demonstrated that Epstein-Barr virus latent membrane protein-2A (LMP2A) induces an enhanced mitochondrial fission to promote cellular migration mediated by Notch signaling in gastric and breast cancer cells^32^. However, the biological roles of interaction between mitochondrial dynamics and Notch signaling in TNBC is still unclear.

In this study, we systematically investigated the alteration of mitochondrial dynamics in TNBC cells and the association with tumor prognosis. Subsequently, functional roles of mitochondrial dynamics in the regulation of cell survival and the underlying molecular mechanisms were explored. Our study provides a novel potential therapeutic strategy for TNBC treatment based on mitochondrial fission machinery.

## Results

### Mitochondrial fission was enhanced in TNBC cells and contributed to poor prognosis of patients

**Fig. 1:**
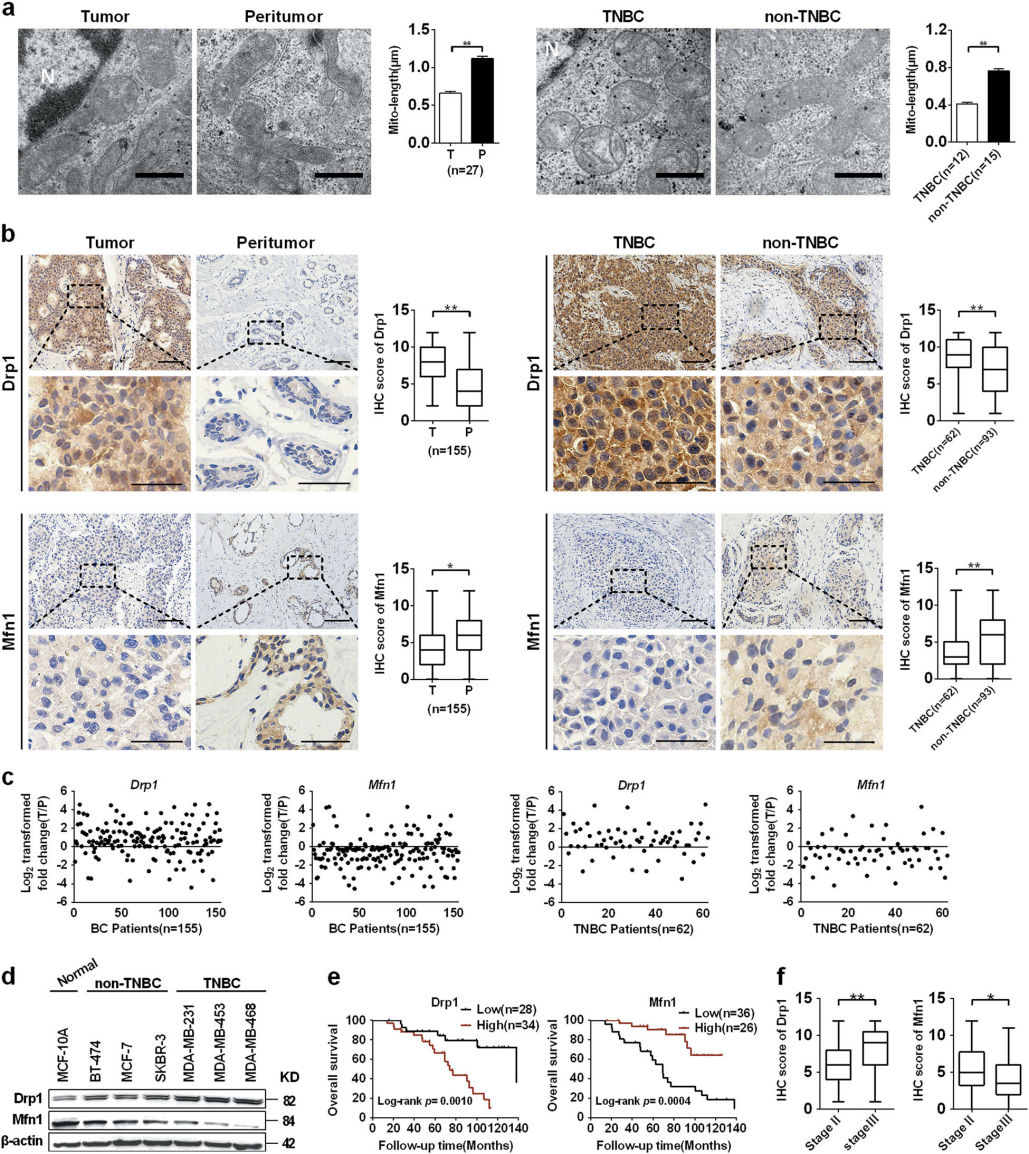
Mitochondrial fission was increased in TNBC and contributes to poor patient prognosis. **a** TEM images of mitochondrial network in paired tissues from TNBC patients (*n* = 12) and non-TNBC patients (*n* = 15). N, nucleus. Scale bar: 0.5 μm. **b** IHC staining images of Drp1 and Mfn1 in paired TNBC tissues (*n* = 62) and non-TNBC tissues (*n* = 93). Scale bar: 100 μm. *P < 0.05; **P < 0.01. **c** The relative mRNA expression ratio (Log_2_ transformed) of tumor/peritumor for Drp1 and Mfn1 measured by qRT-PCR in 155 pairs of BC tissues. **d** Western blot for expression levels of Drp1 and Mfn1 in human breast cell lines. **e** Kaplan–Meier curve of overall survival in TNBC patients with different expression levels of Drp1 and Mfn1. **f** The protein expression levels of Drp1 and Mfn1 in tissue chips of stage II/III TNBC patients (*n* = 62) were evaluated based on score of IHC staining

To explore the functional roles of mitochondrial dynamics in TNBC cells, we first examined the mitochondrial morphology in 27 breast cancer tissues and their adjacent normal tissues using transmission electron microscopy (TEM). As shown in Fig. 1a, mitochondrial length in BC tissues was significantly shorter than that in adjacent non-tumor tissues. Moreover, mitochondria were much shorter in TNBC tissues than that in non-TNBC tissues. We further analyzed the expression of Drp1 and Mfn1, the two critical molecules for mitochondrial dynamics regulation, using immunohistochemical staining. As shown in Fig. 1b, mitochondrial fission mediator Drp1 was significantly increased while mitochondrial fusion protein Mitofusin1 (Mfn1) was remarkably downregulated in BC tissues. Moreover, we noted a much higher Drp1 and lower Mfn1 expression in TNBC than in non-TNBC tissues. In line with IHC scores, real-time PCR analysis showed similar mRNA expression pattern of Drp1 and Mfn1 in both BC and TNBC tissues (Fig. 1c). Consistently, our Western blot analysis also showed a higher Drp1 and lower Mfn1 in TNBC cell lines than in non-TNBC cell lines (Fig. 1d). Moreover, we analyzed the association of mitochondrial dynamic mediator with TNBC prognosis. As shown in Fig. 1e, patients with high Drp1 expression or low Mfn1 expression had a significantly poorer overall survival (Fig. 1e). In addition, patients at stage III TNBC showed higher Drp1 and lower Mfn1 expression than patients at stage II TNBC (Fig. 1f), suggesting that mitochondrial dynamics contribute to TNBC progression.

### Drp1 and Mfn1 modulated mitochondrial dynamics in TNBC cells

**Fig. 2:**
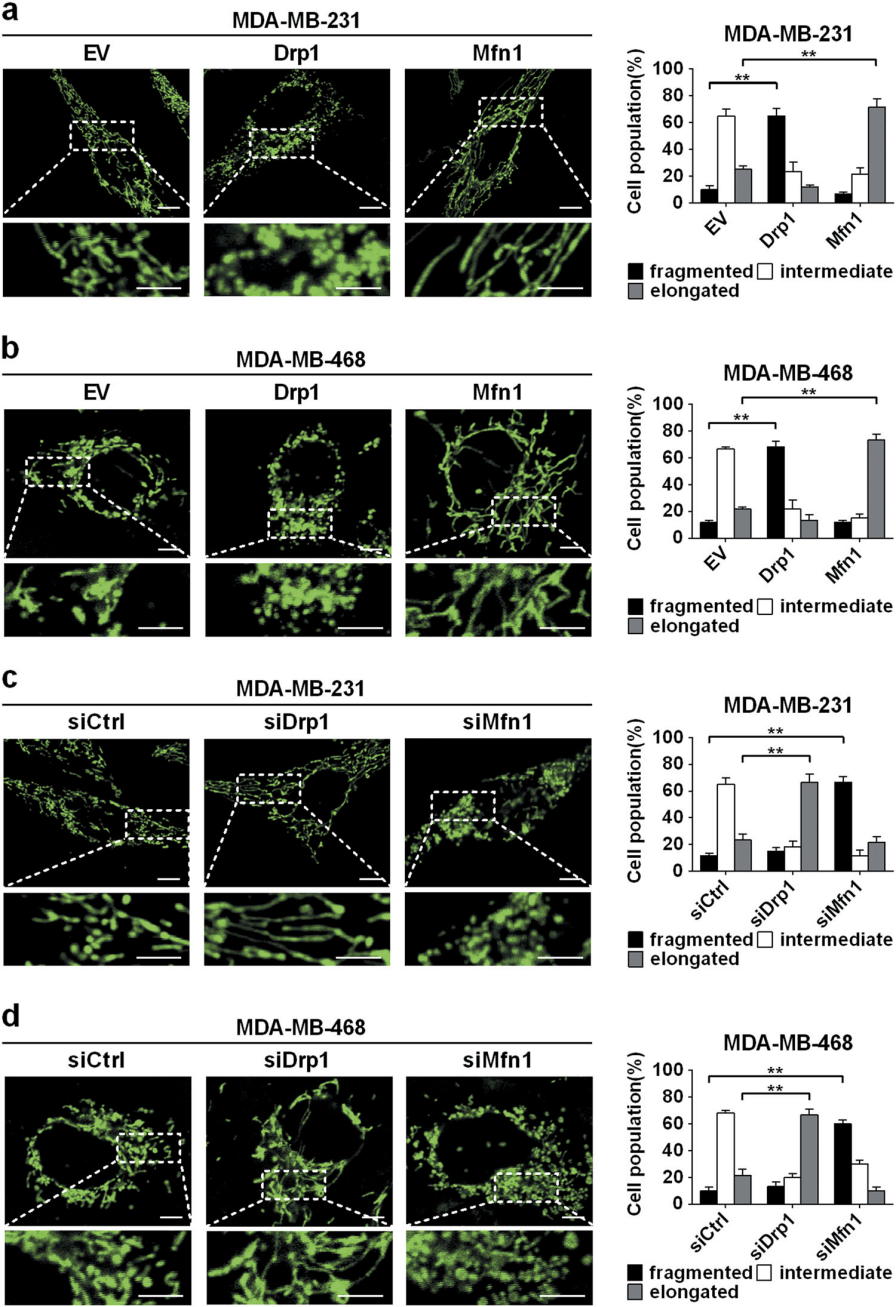
Mitochondrial dynamics was remodeled by regulating Drp1 and Mfn1 in TNBC cells. After TNBC cells (MDA-MB-231 and MDA-MB-468) were transfected with expression vectors or siRNA for 48 h, MitoTracker green staining and confocal microscopy analysis of mitochondrial network were performed as described EV, Drp1, Mfn1, siCtrl, siDrp1, siMfn1. Scale bars: 5 μm. *P < 0.05; **P < 0.01

As Drp1 and Mfn1 were key mediators of mitochondrial dynamics, we assessed the effects of Drp1 and Mfn1 on mitochondrial dynamics in TNBC cell lines MDA-MB-231 and MDA-MB-468. As shown in Supplementary Figure S1 and Fig. 2a, b, Drp1 overexpression significantly increased mitochondrial fission in both cell lines, as indicated by shortened mitochondrial length. As Drp1 has to be phosphorylated to be active as effector of the mitochondrial fission machinery, we further check the level of p-Drp1, which was positively related to the level of Drp1 (Supplementary Figure S1c). We also evaluated the expression of p-Drp1 using immunohistochemical staining. As shown in Supplementary Fig. 1e, we noted a much higher p-Drp1 expression in TNBC than in non-TNBC tissues, which was in line with those of Drp1 expression.

In contrast, Mfn1 overexpression increased mitochondrial fusion in TNBC cells, as indicated by elongated mitochondrial length. Silencing Drp1 or Mfn1 by siRNA showed opposite effects on mitochondrial dynamics in both cell lines (Supplementary Figure S1, Fig. 2c, d). These results indicated that mitochondrial fission and fusion could be remodeled by the alteration of Drp1 or Mfn1 expression in TNBC cells.

### Mitochondrial fission promoted TNBC cell survival

**Fig. 3:**
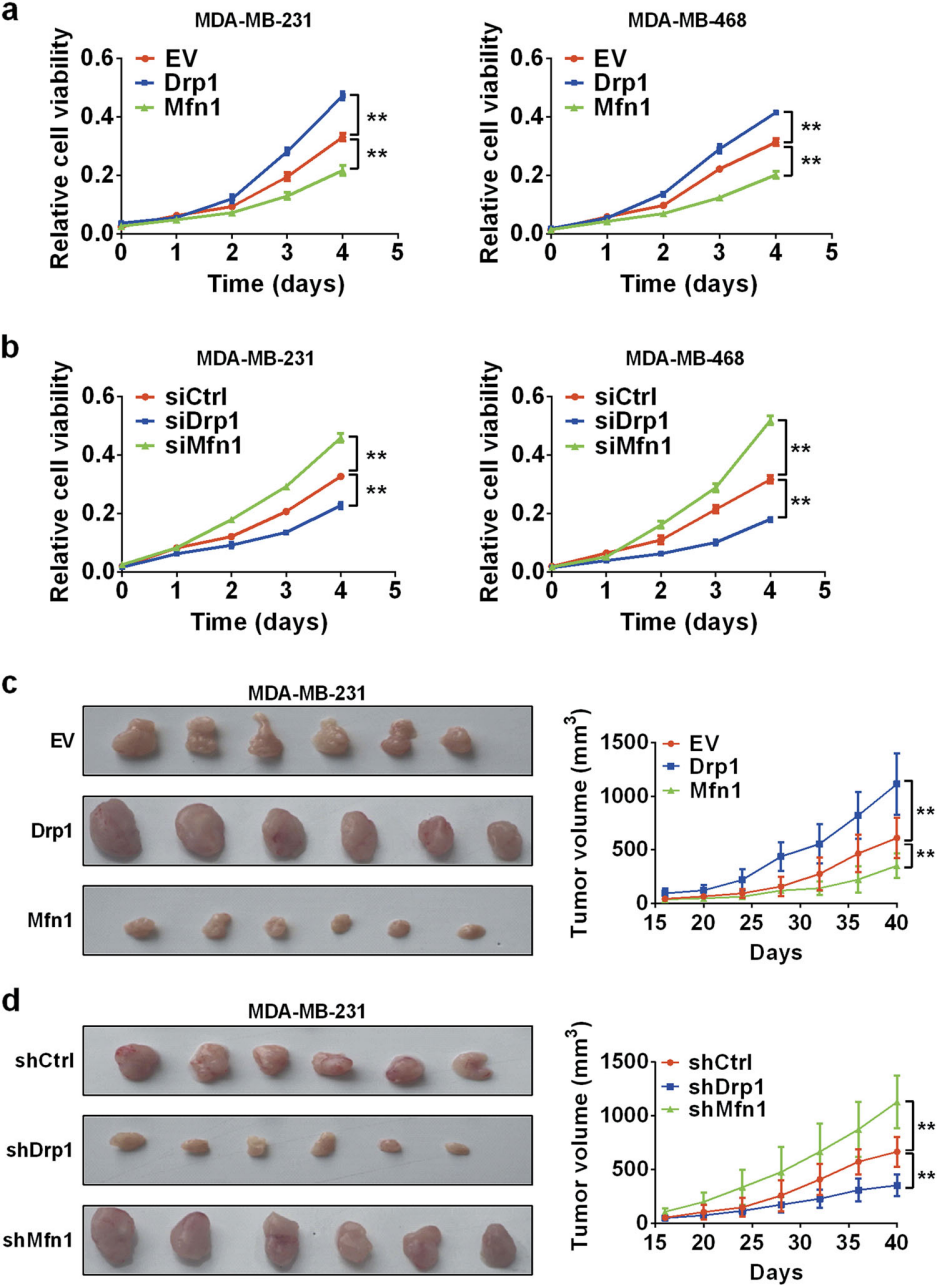
Mitochondrial fission promoted cell survival in TNBC cells. **a**, **b** MTS cell viability was analyzed in TNBC cells treated as indicated. **c**, **d** Tumor growth curves of subcutaneous xenograft tumor model and dissected tumors from sacrificed mice

Next, we investigated the effect of mitochondrial dynamics on TNBC cell survival. MTS assays showed that Drp1 silencing or Mfn1 overexpression was associated with a lower survival rate when compared to the control groups in both cell lines, and vice versa (Fig. 3a, b). Additionally, we examined the impact of unbalanced mitochondrial fission and fusion on tumor growth in vivo (Supplementary Figure S2). Similarly, there was a significant decrease in growth capacity of xenograft tumors developed from MDA-MB-231 cells with stable Drp1 silencing or Mfn1 overexpression, whereas the growth capacity of xenograft tumors developed from cells with stable Drp1 overexpression or Mfn1 silencing were significantly increased than that in control xenograft tumors (Fig. 3c, d). These results revealed that Drp1-mediated mitochondrial fission promoted TNBC cell survival, whereas Mfn1-mediated mitochondrial fusion had an opposite effect.

### Mitochondrial fission promoted TNBC cell growth by inhibiting apoptosis and accelerating cell proliferation in vivo and in vitro

**Fig. 4:**
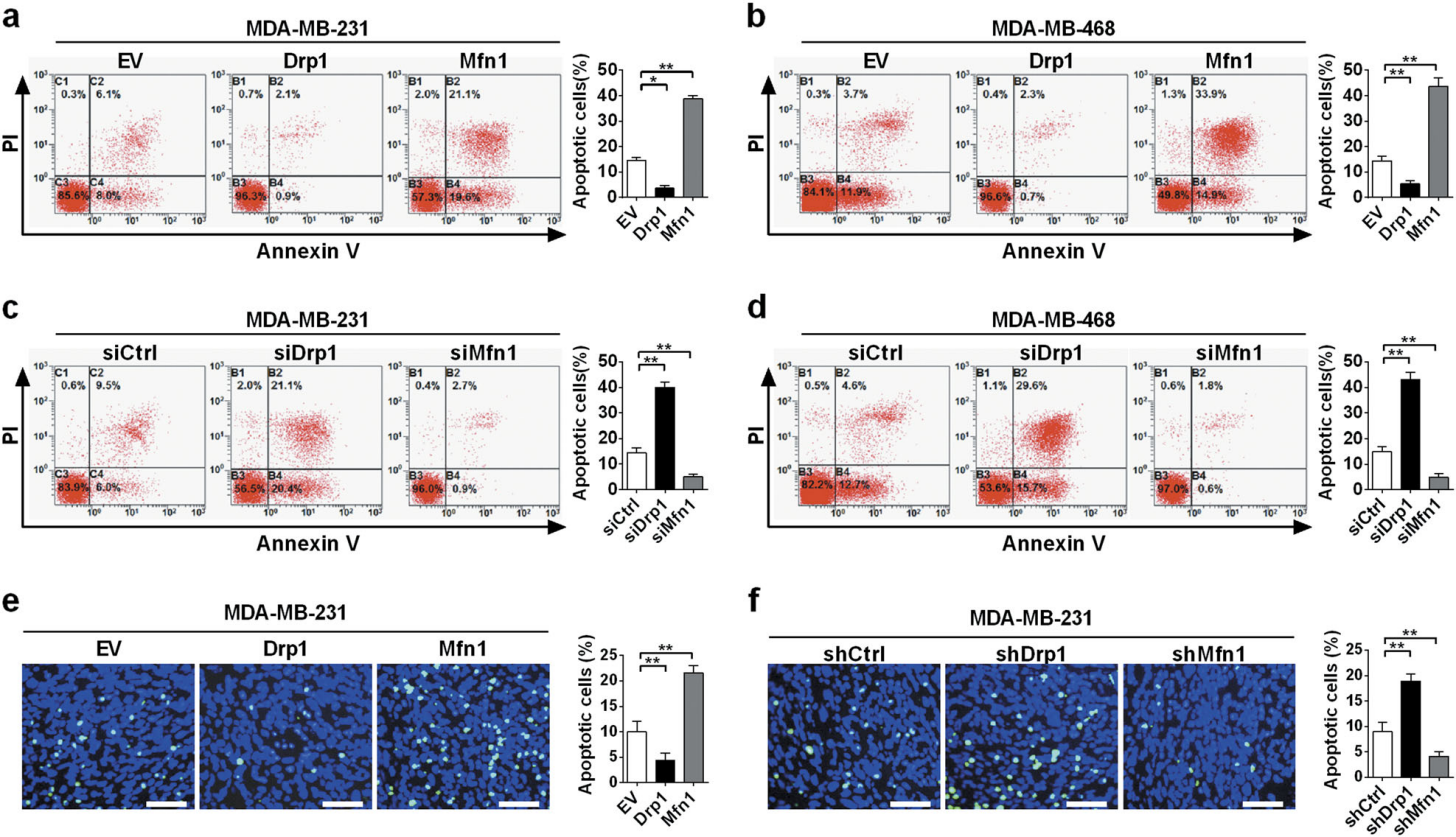
Mitochondrial fission inhibited TNBC cell apoptosis. **a**–**d** Apoptosis of TNBC cells analyzed by flow cytometry 48 h after siRNAs or plasmid transfection. Apoptosis was induced by 1 μM thapsigargin for 24 h. **e**, **f** TUNEL staining in tumor tissues dissected from xenograft tumors developed from MDA-MB-231 cells with different mitochondrial dynamic status. Green, TUNEL-positive nucleus; Blue, Hochest33342. Scale bar: 50 μm. *P < 0.05; **P *<* 0.01

Given our finding that Drp1-mediated mitochondrial fission promoted TNBC cell survival, we further assessed the functional role of mitochondrial dynamics in apoptosis and proliferation of TNBC cells. Our results revealed that the apoptotic rate was significantly lower in TNBC cells with Drp1 overexpression or Mfn1 knockdown than that in control cells. Besides, our data also showed that Drp1 knockdown or Mfn1 overexpression remarkably induced apoptosis in Thapsigargin-treated and C2-ceramide-treated TNBC cells (Fig. 4a–d and Supplementary Figure S5a-d). In addition, TUNEL staining in xenograft tumor tissues confirmed the effects of mitochondrial dynamics on TNBC cell apoptosis (Fig. 4e, f).

**Fig. 5:**
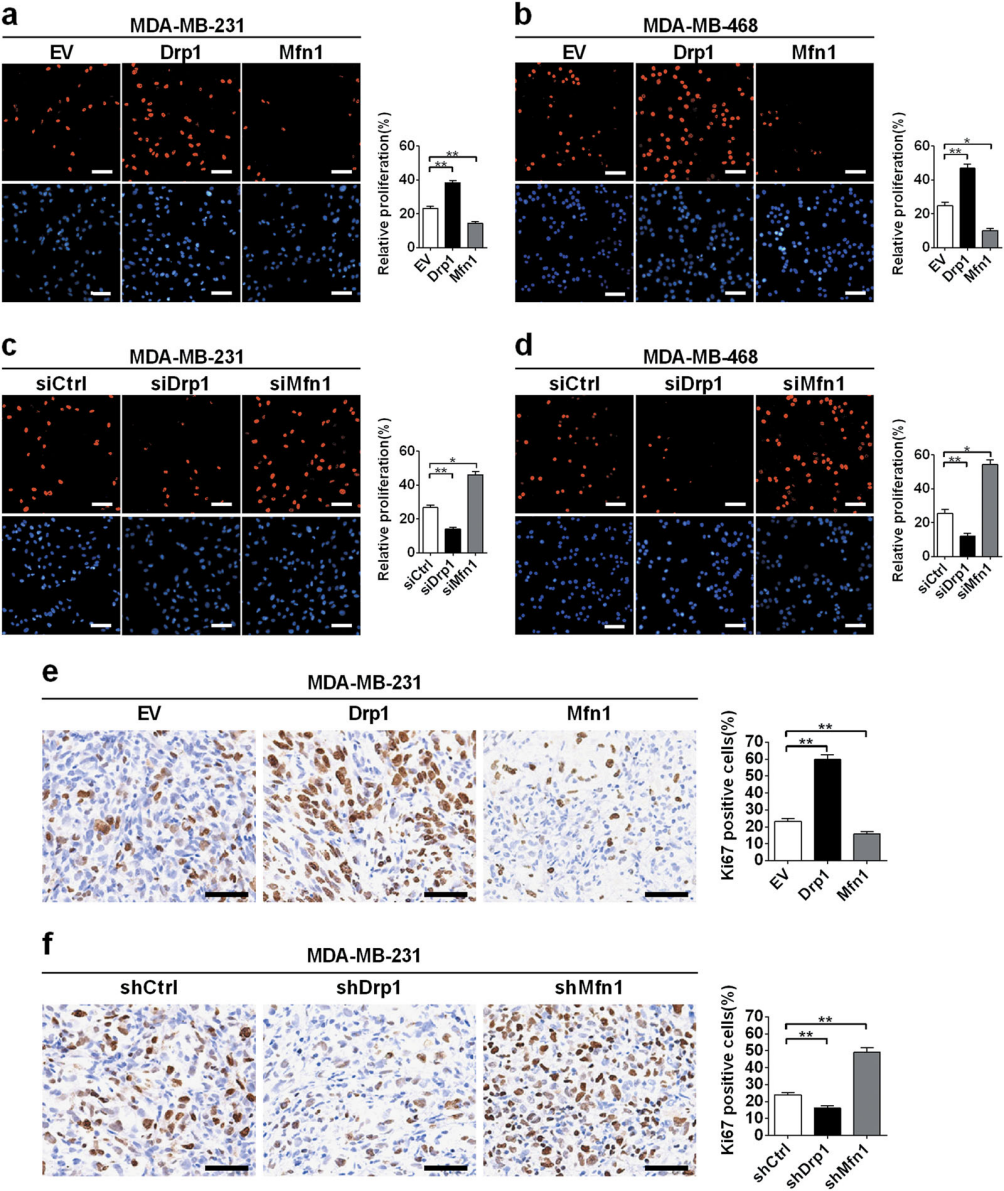
Mitochondrial fission promoted TNBC cell proliferation. **a**–**d** Proliferation of TNBC cells evaluated by EdU incorporation analysis after different treatment. Data was presented as mean ± SEM, *n* = 3. *P < 0.05; **P < 0.01. **e**, **f** Representative IHC staining images of Ki67 in tumor tissues dissected from xenograft tumor treated as indicated. Scale bar: 50 μm. *P < 0.05; **P < 0.01

We next explored the effect of mitochondrial fission and fusion on TNBC cell proliferation. EdU incorporation assay indicated that the proliferation of TNBC cells with Drp1 overexpression or Mfn1 knockdown was obviously increased. In contrast, Drp1 knockdown or Mfn1 overexpression exhibited an opposite effect in TNBC cells (Fig. 5a–d). In xenograft tumor tissues, immunohistochemical staining of Ki67 (a nuclear proliferation antigen) also supported the functional role of mitochondrial fission in promoting TNBC growth (Fig. 5e, f). Overall, our data indicated that Drp1-mediated mitochondrial fission promoted cell growth by inhibiting apoptosis and accelerating cell proliferation in TNBC.

### A positive feedback loop existed between mitochondrial fission and Notch signaling in TNBC cells

**Fig. 6:**
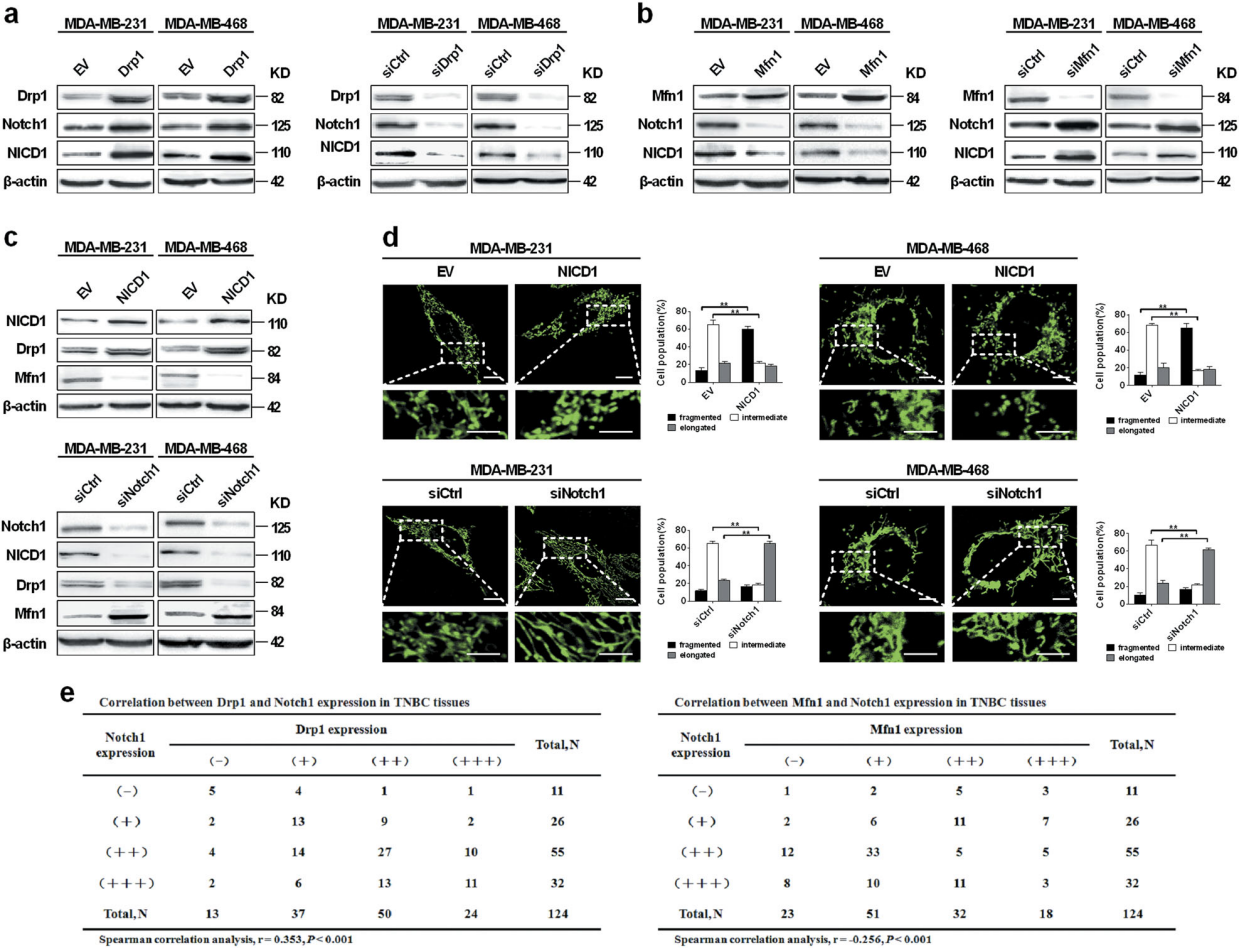
A positive feedback loop between mitochondrial fission and notch signaling. **a**–**c** Western blot analysis for Drp1, Mfn1, Notch1 and NICD1 in TNBC cells with different treatment. **d** Changes of mitochondrial dynamics in TNBC cells after Notch signaling alterations. Scale bar: 5 μm. *P < 0.05; **P < 0.01. **e** Correlation between IHC scores of Notch1 and Drp1 or Mfn1 in 124 TNBC tissues

Previous studies have demonstrated a link between mitochondria and Notch signaling, in which genetic ablation of Drp1 is accompanied by Notch inactivation, leading to the inhibition of follicle cell differentiation^30^. Notch pathway has been previously proved to play a critical role in TNBC cell survival^33,34^. Therefore, we explored whether there was a crosstalk between mitochondrial dynamics and Notch signaling in TNBC. As shown in Fig. 6a, b, the level of both Notch1 and activated form of Notch1 (NICD1, also known as Notch1 intracellular domain) were upregulated in TNBC cells after Drp1 overexpression or Mfn1 knockdown. In contrast, Drp1 knockdown or Mfn1 overexpression in TNBC cells induced an opposite effect. Reciprocally, NICD1 overexpression significantly enhanced mitochondrial fission by increasing the expression of Drp1 and suppressing Mfn1 expression (Fig. 6c, d). In contrast, Notch1 silencing by siRNA showed opposite effects on the expression of Drp1 and Mfn1, as well as mitochondrial dynamics. We further assessed the association between expression of Notch1 and mitochondrial dynamic mediators. As shown in Fig. 6e, there was a positive correlation between IHC scores of Drp1 and Notch1 (*ρ* = 0.353, *P* < 0.001) and a notable negative correlation between Mfn1 and Notch1 (*ρ* = −0.256, *P* < 0.001). Collectively, these results indicated that mitochondrial fission and Notch signaling could interact with each other reciprocally to form a positive feedback loop in TNBC cells.

### Positive feedback loop between mitochondrial fission and Notch signaling regulated cell apoptosis and proliferation in TNBC cells

**Fig. 7:**
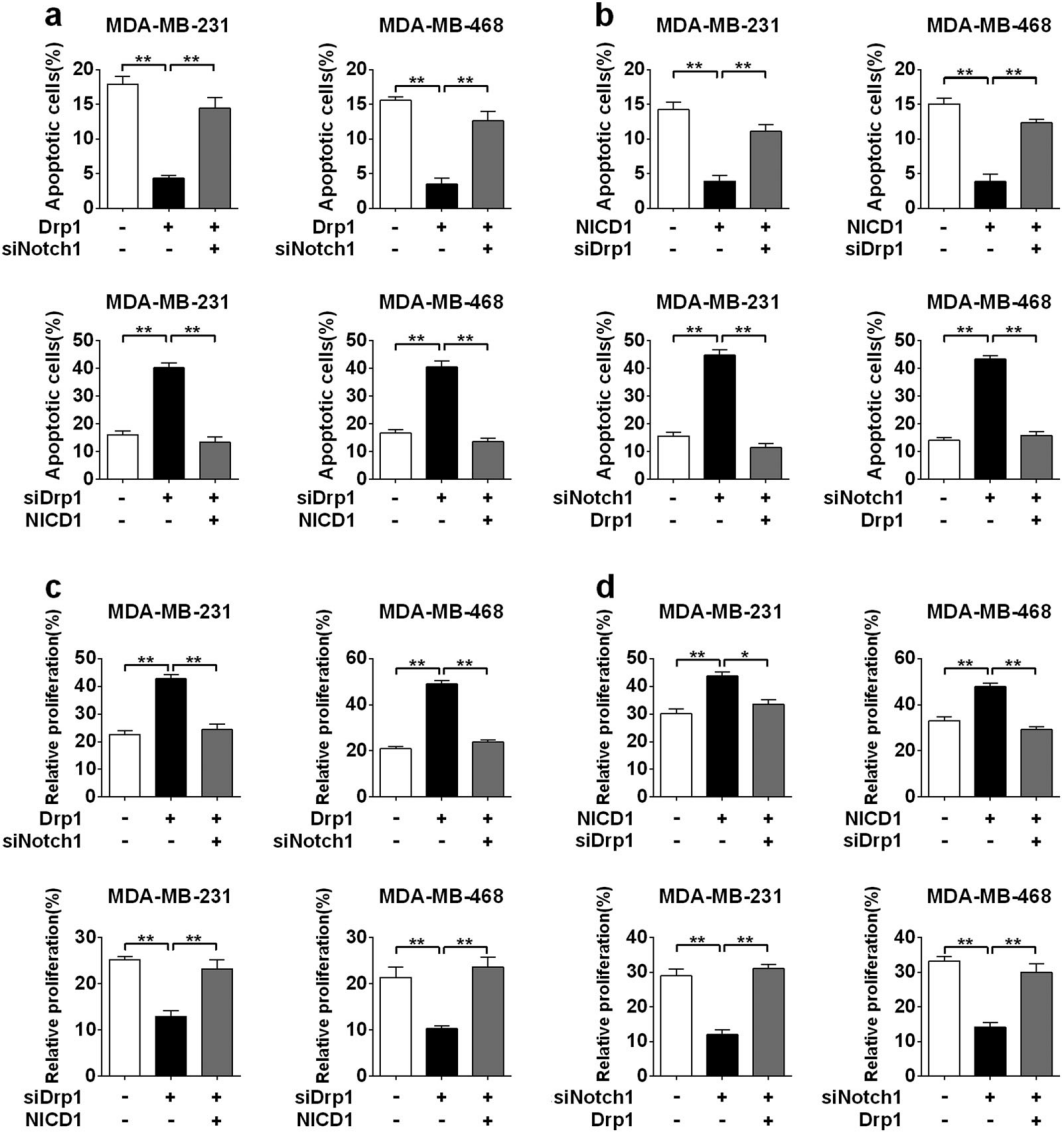
Notch-mitochondrial fission positive feedback loop regulated cell apoptosis and proliferation in TNBC. **a**, **b** Thapsigargin-induced apoptosis analyzed by flow cytometry in TNBC cells after transfection with different siRNAs or plasmids. **c**, **d** Cell proliferation determined by EdU analysis in TNBC cells 48 h after transfection with different expression vectors or siRNAs. Data was presented as mean ± SEM, *n* = 3. *P < 0.05; **P < 0.01

We further determined the effect of the positive feedback loop between mitochondrial fission and Notch signaling on TNBC cell survival. Our data indicated that Drp1 overexpression inhibited apoptosis, which could be eliminated by Notch1 knockdown, while the activation of Notch signaling by NICD1 overexpression could rescue Drp1 silencing-induced apoptosis in both cell lines (Supplementary Figure S3a, Fig. 7a). On the other hand, NICD1 overexpression reduced apoptosis of TNBC cells, which could be reversed by Drp1 knockdown, whereas Notch silencing-induced TNBC cell apoptosis could be rescued by Drp1 overexpression (Supplementary Figure S3b, Fig. 7b). Furthermore, Notch1 silencing antagonized the proliferative effects of Drp1 overexpression, while NICD1 overexpression restored the proliferative capacity after Drp1 silencing (Supplementary Figure S4a, Fig. 7c). On the other hand, Notch signaling-mediated cell proliferation also relied on Drp1 expression and further analysis showed that the increased proliferative activity of TNBC cell with Drp1 or NICD1 overexpression can be significantly decreased by Notch1 or Drp1 knockdown (Supplementary Figure S4b, Fig. 7d). Similarly, the decreased proliferation of TNBC cell with Drp1 or Notch1 knockdown could be recovered by overexpression of Drp1 or NICD1, respectively (Supplementary Figure S4, Fig. 7c, d). These data suggested a critical role between mitochondrial fission and Notch signaling in TNBC cell survival.

### Positive feedback loop between mitochondrial fission and Notch signaling upregulated survivin to promote TNBC cell survival

**Fig. 8:**
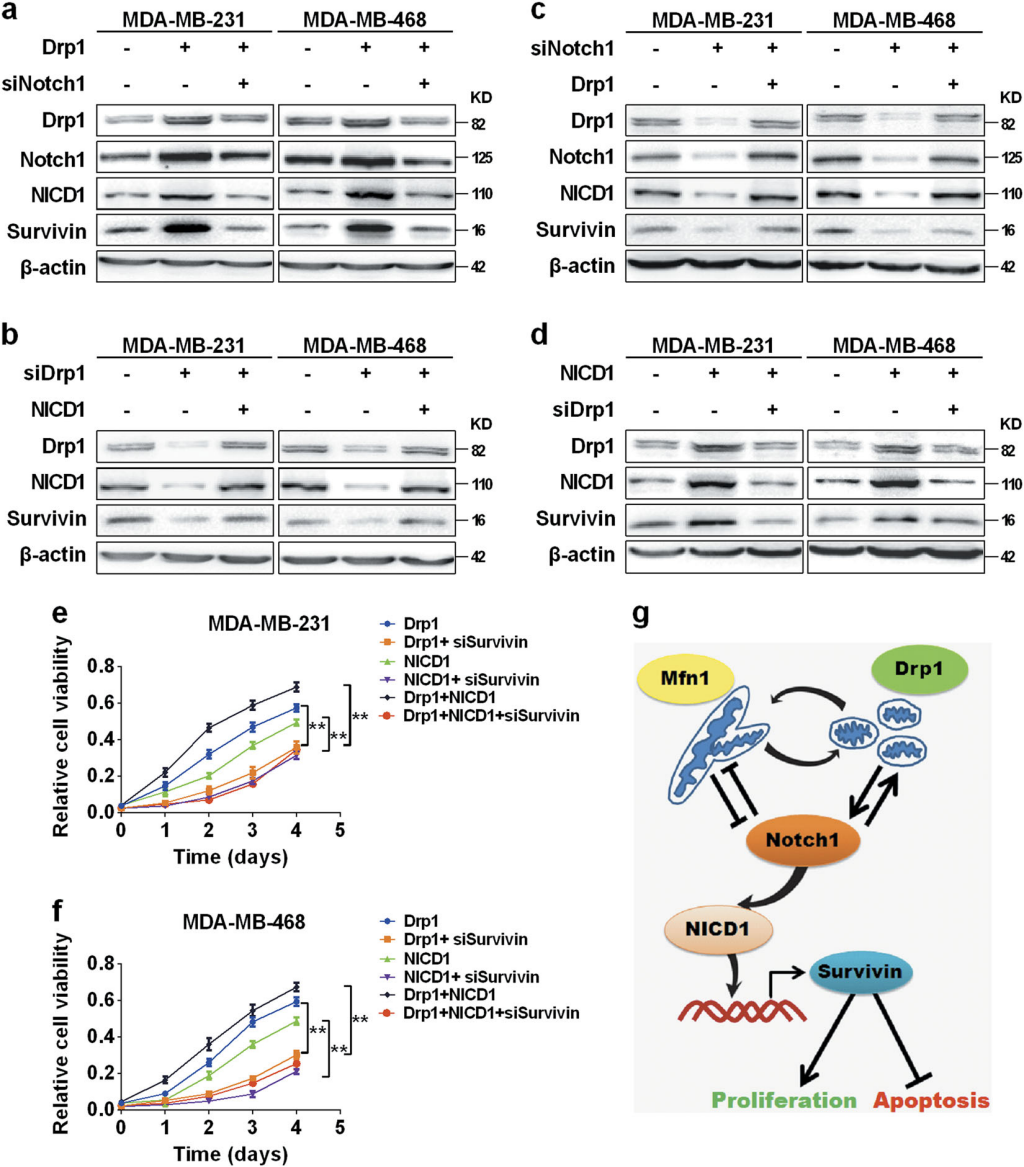
Notch-mitochondrial fission positive feedback loop-induced survivin expression promoted TNBC cell survival. **a**–**d** Western blot analysis for Drp1, Notch1, NICD1 and survivin in TNBC cells with transfection as indicated. **e**, **f** Cell viability analyzed by MTS in TNBC cells transfected with expression vectors or siRNAs as indicated. *P < 0.05; **P *<* 0.01. **g** Schematic depicting the effect of the feedback loop between mitochondrial dynamics and Notch signaling on TNBC cell survival and underlying mechanism

Previous evidence has indicated that Notch signaling pathway is closely associated with survivin^35^, which is important for TNBC cell growth^36^. Therefore, we investigated whether survivin expression was modulated by the feedback loop between mitochondrial fission and Notch signaling in TNBC cells. As shown in Fig. 8a, Drp1 overexpression elevated the expression of survivin, which could be inhibited by Notch silencing. Further, survivin overexpression elevated the level of Drp1 and influenced the Drp1-mediated fission, along with alterations of mitochondrial morphology and function (Supplementary Figure S6a–c). In contrast, Drp1 silencing suppressed survivin expression, which could be reversed by NICD1 overexpression (Fig. 8b). On the other hand, Notch1 silencing-induced survivin downregulation could be reversed by Drp1 overexpression, whereas NICD1-mediated survivin expression could be inhibited by Drp1 silencing (Fig. 8c, d). In addition, MTS assays indicated that TNBC cells with upregulation of both Drp1 and NICD1 exhibited the highest cell viability, whereas those cells with upregulation of either Drp1 or NICD1 exhibited a moderate cell viability. In contrast, silencing of survivin significantly reversed the survival-promoting effect of Drp1 and NICD1 (Fig. 8e, f). Collectively, our data demonstrated that crosstalk between mitochondrial fission and Notch signaling induced the expression of survivin, which ultimately promoted TNBC cell survival (Fig. 8f).

## Discussion

A series of previous studies have demonstrated that abnormal mitochondrial dynamics, a process controlled by a family of GTP-dependent dynamin related proteins such as Drp1 and Mfn1, is implicated in the development and progression of many human cancers^25^. The increased mitochondrial fission, which is mediated by upregulated Drp1 and downregulated Mfn2, has been observed in lung cancer cells^27^. Similarly, we observed the increased mitochondrial fission in TNBC cells, which was mediated by upregulated Drp1 and/or downregulated Mfn1. In recent years, accumulating data have reported the roles of mitochondrial fusion and fission in tumor cell survival^37,38^. For example, the inhibition of Erk2-mediated phosphorylation of Drp1 Ser616 is sufficient to block pancreatic cancer cell growth^39^. Moreover, Mfn2 overexpression suppresses the proliferation of gastric cancer cell through P21 and PI3K/Akt signaling^40^. In addition, Qian et al. have demonstrated that mitochondrial hyperfusion results in G2/M arrest and cell death in breast cancer cells^41^. Consistent with these reports, our study found that increased mitochondrial fission markedly promoted TNBC cell survival through promoting cell proliferation and inhibiting cell apoptosis while increased mitochondrial fusion had an inhibitory effect on TNBC cell survival. Furthermore, the poor clinical outcomes and higher disease stage in TNBC patients with increased mitochondrial fission highlighted the clinical significance of our findings. Consistently, a previous study has reported that CDK5-mediated Drp1 activation significantly promotes mitochondrial fission and correlates with poor prognosis in brain tumor^42^.

A series of signaling pathways involved in the regulation of mitochondrial dynamic^43^, such as phosphoinositide 3-kinase (PI3K) and mitogen-activated protein kinase (MAPK), have been extensively studied in cancer cells. Notch signaling has also been reported to be closely linked to mitochondrial dynamics^29,30^. Emerging evidence has revealed that Notch signaling pathway is activated in breast cancer cells and contributes to the cancer progression^6,44–46^. Moreover, our results demonstrated that mitochondrial fission was increased by the activation of Notch pathway in TNBC cells. Consistently, recent studies indicate that Notch signaling is another novel factor that affects mitochondrial shape. For example, by inhibiting mitochondrial fission and Bax oligomerization^29^, the activated Notch signaling regulates cell survival through the interaction with Mfns. The inhibition of Notch signaling mediated by the viral latent membrane protein 2A significantly decreases the mitochondrial fission by reducing Drp1 expression^32^. In addition, Notch signaling also recruits Akt pathway to protect mitochondria from apoptosis by activating Mfns to induce mitochondrial fusion^29^.

Moreover, previous studies have reported that the crosstalk between nuclear signaling and mitochondria is bidirectional. Recent observations suggest the possible mechanisms by which mitochondrial dynamics affects Notch signaling. Mfn/OPA downregulation in mouse embryonic stem cells (ESCs) during cardiomyocyte development results in increased cytoplasmic Ca^2+^ load and the activity of calcineurin. Therefore, Notch signaling is activated by calcineurin, which results in the increase of active NICD in nucleus^31^. In a different model, reactive oxygen species (ROS)^47^ acts as the signal from mitochondria to affect Notch signaling. Consistent with these reports, our data showed that mitochondrial fission promoted Notch signaling activation by upregulating the Notch1 and NICD1 in TNBC cells. These findings demonstrate a positive feedback loop between mitochondrial fission and Notch signaling.

Survivin, a member of IAP family, acts as suppressor of apoptosis in many cancers, including breast and lung cancer^48,49^. Accumulating evidence has indicated that survivin expression is upregulated by Notch signaling. The activation of Notch1 signaling promotes the upregulation of survivin and results in the inhibition of apoptosis in ER-negative breast cancer cells^36^. Consistently, our results suggested that the disruption of the mitochondrial fission/Notch1 feedback may inhibit the survivin–mediated TNBC development. In a previous study in lung cancer cells, it has been reported that the activation of Notch1 signaling facilitate the binding of RBP-Jκ to the promoter of survivin, thus resulting in the transcriptional activation of survivin^35^. Therefore, it is conceivable that mitochondrial fission-mediated Notch signaling activation may upregulate survinvin expression in a similar transcriptional manner in TNBC cells, which needs to be further investigated.

In summary, our study identified the contribution of increased mitochondrial fission to the progression of BC, especially TNBC. Moreover, the increased mitochondrial fission enhanced the activity of Notch1 signaling, which in turn promoted mitochondrial fission via Drp1 upregulation and Mfn1 downregulation, suggesting a positive feedback loop between mitochondrial fission and Notch signaling in TNBC cells. Moreover, such positive feedback loop enhanced the apoptotic resistance and survival of cells, thus promoting the progression of TNBC. Our data provide a novel insight into the relationship between Notch signaling and mitochondrial dynamics and their roles in TNBC.

## Materials and methods

### Cell culture and tissue collection

Human normal breast cell lines MCF-10A, breast cancer cell lines MCF-7, MDA-MB-231, MDA-MB-453, and MDA-MB-468 were routinely cultured in DMEM medium. Breast cancer cell lines BT-474 and SKBR-3 were routinely cultured in RPMI-1640 medium. And all these cell lines were purchased from Shanghai Cell Bank of Chinese Academy of Sciences (Shanghai, China). One hundred and fifty five tissue samples (including TNBC and non-TNBC tissue samples) from breast cancer patients were collected at Xijing Hospital, Fourth Military Medical University in Xi’an, China. The demographic information, clinical and follow-up data of these 155 patients were collected by clinical specialists or trained interviewers. The latest follow-up date was July 2014. The follow-up duration ranged from 14.1 to 138.2 mo (the median follow-up duration was 69.4 mo. The time from surgery to TNBC specific death was defined as overall survival. The informed consent was obtained from all participants study and our study was approved by the Ethics Committee of the Fourth Military Medical University. Tissue chips of stage II/III TNBC patients were purchased from Alena Biotechnology (Xi’an, China).

### Plasmid and siRNA

For the overexpression of Drp1, Mfn1 or NICD1, the coding sequences of Drp1, Mfn1 or NICD1 was amplified from cDNA derived from HEK293 cells using primers listed in Supplementary Table 1 and cloned into the pcDNA™3.1(+) vector (Invitrogen). All siRNAs were synthesized by GenePharma (Shanghai, China). The sequences of siRNAs for Drp1, Mfn1, Notch1, and Survivin are provided in Supplementary Table 1. For the generation of shRNA expression vectors, a small hairpin RNA (shRNA) containing negative control sequence or specific sequences targeting the human Drp1 or Mfn1 mRNA sequence was cloned into the pSilencer™ 3.1-H1 puro vector (Ambion).

### Antibodies and reagents

The primary antibodies used in this study and their working concentration were listed in Supplementary Table 2. Thapsigargin (TG, an endoplasmic reticular Ca2^+^-ATPase inhibitor) was purchased from Sigma-Aldrich (St. Louis, MO, USA). C2-ceramide was purchased from Enzo Life Sciences (USA)

### Mitochondrial network imaging by electron microscopy and confocal microscopy

Conventional transmission electron microscopy analysis was performed as described previously^50^. Thin sections were analyzed with a Tecnai G2 electron microscope (FEI, cHillsboro, Oregon) at 43000 magnifications. The fluorescent dye MitoTracker green FM (Molecular Probes, Invitrogen) was used to monitor mitochondrial morphology in living cells according to the manufacturer’s instructions. Then cells were viewed with an Olympus FV 1000 laser-scanning confocal microscope (Olympus Corporation, Tokyo, Japan). For morphometric analysis, the length of mitochondria was measured using the ImageJ software (NIH, Bethesda, MD, USA). In addition, the number of mitochondria was counted and averaged in 50 cells per sample.

### Quantitative real-time reverse transcription PCR (qRT-PCR)

Total RNA extraction, complementary DNA synthesis, qRT-PCR reactions were performed according to the manufacturer’s instructions. Primer sequences used in this study were provided in Supplementary Table 1.

### Western blot and immunohistochemistry

For Western blot analysis, cell lysates were subjected to SDS–PAGE and transferred to PVDF membranes. The blots were probed with indicated primary antibodies and followed by secondary antibodies. Process of tissues for IHC and quantification of IHC staining score was performed as previously described^51^.

### Nude mice xenograft model

Female BALB/c nude mice at 6 weeks of age were divided into groups randomly. Stably transfected MDA-MB-231 cells (1 × 10^7^cells/mouse) were suspended in matrigel matrix and inoculated subcutaneously in nude mice. Tumor length (l) and width (w) was measured using vernier calipers every 4 days. Tumor volumes were assessed according to the formula (l × W^2^)/2. After 40 days, mice were euthanized by CO_2_ asphyxiation followed by cervical dislocation and the tumor nodules were harvested and photographed. This study was approved by the Ethics Committee of the Fourth Military Medical University for animal research.

### Cell apoptosis and viability assays

Cell apoptosis was determined with an Annexin V-FITC/PI Apoptosis Detection Kit (BestBio, Shanghai, China) as the manufacturers’ instructions described. TNBC cells seeded in 6-well plates were collected and resuspended with 400 μL binding buffer. After adding 5 μL Annexin 5-FITC and 5 μL PI, cells were mixed and incubated at room temperature in the dark for 15 min. The samples were analyzed with a flow cytometer (Beckman, Fullerton, CA, USA). For analysis of apoptosis in xenograft tissues, terminal deoxynucleotidyl transferase-mediated dUTP nick-end labeling (TUNEL) assay (Roche Applied Science,) was performed according to the manufacturer’s protocol. Images of TUNEL and DAPI-stained sections were obtained by a fluorescence microscope (DM5000B; Leica, Heerbrugg, Switzerland). Results were expressed as the mean number of TUNEL-positive apoptotic TNBC cells in each field. MTS assay (Promega Corporation, G3581) was performed for cell viability according to the manufacturer’s instructions. Simply, 1.5 × 10^3^ cells were plated in each well of a 96-well culture plate. After 24 h, cell viability was measured by addition of 10 μL of MTS (0.2%)-PMS (0.092%; phenazine methosulfate, 20:1) solution and incubation for 1 h. The microplates were read in a spectrophotometer at a wavelength of 490 nm. Each sample was analyzed in triplicate.

### Cell proliferation assay

Cell proliferation was determined with Cell-Light^TM^ EdU DNA Cell Proliferation Kit (Ribobio, China). Briefly, cells were incubated with EdU reagent 48 h after transfection. Then permeabilization buffer was added to cells followed by washing with PBS. After stained with Apollo solution for 30 min, cells were observed by using fluorescence microscopy. Experiments were repeated at least three times in duplicates.

### Statistical analysis

Experiments were performed independently at least three times where appropriate. SPSS 17.0 software (SPSS, Chicago, IL, USA) was used for all statistical analyses, and *P* values *<* 0.05 was considered statistically significant. Unpaired *t*-test was used for comparisons between two groups where appropriate. For prognosis analysis, variables (the IHC score and expression ratio of Drp1 and Mfn1) were divided into high or low level by the median value for further analysis. The log-rank test and Kaplan–Meier survival curve were used to distinguish subgroup patients with different survival. Correlations between measured variables were tested by Spearman rank correlation analysis.

## Electronic supplementary material

Figure S1 related to fig 2

Figure S2 related to fig 3

Figure S3 related to fig 7

Figure S4 related to fig 7

Figure S5 related to fig 4

Figure S6 related to fig.8

Supplementary Information
