# Supplementary figures and images for: Positive feedback loop between mitochondrial fission and Notch signaling promotes survivin-mediated survival of TNBC cells
Source: Cell Death Dis. 2018 Oct 15;9(11):1050. doi: 10.1038/s41419-018-1083-y (PMC6189045; doi:10.1038/s41419-018-1083-y)

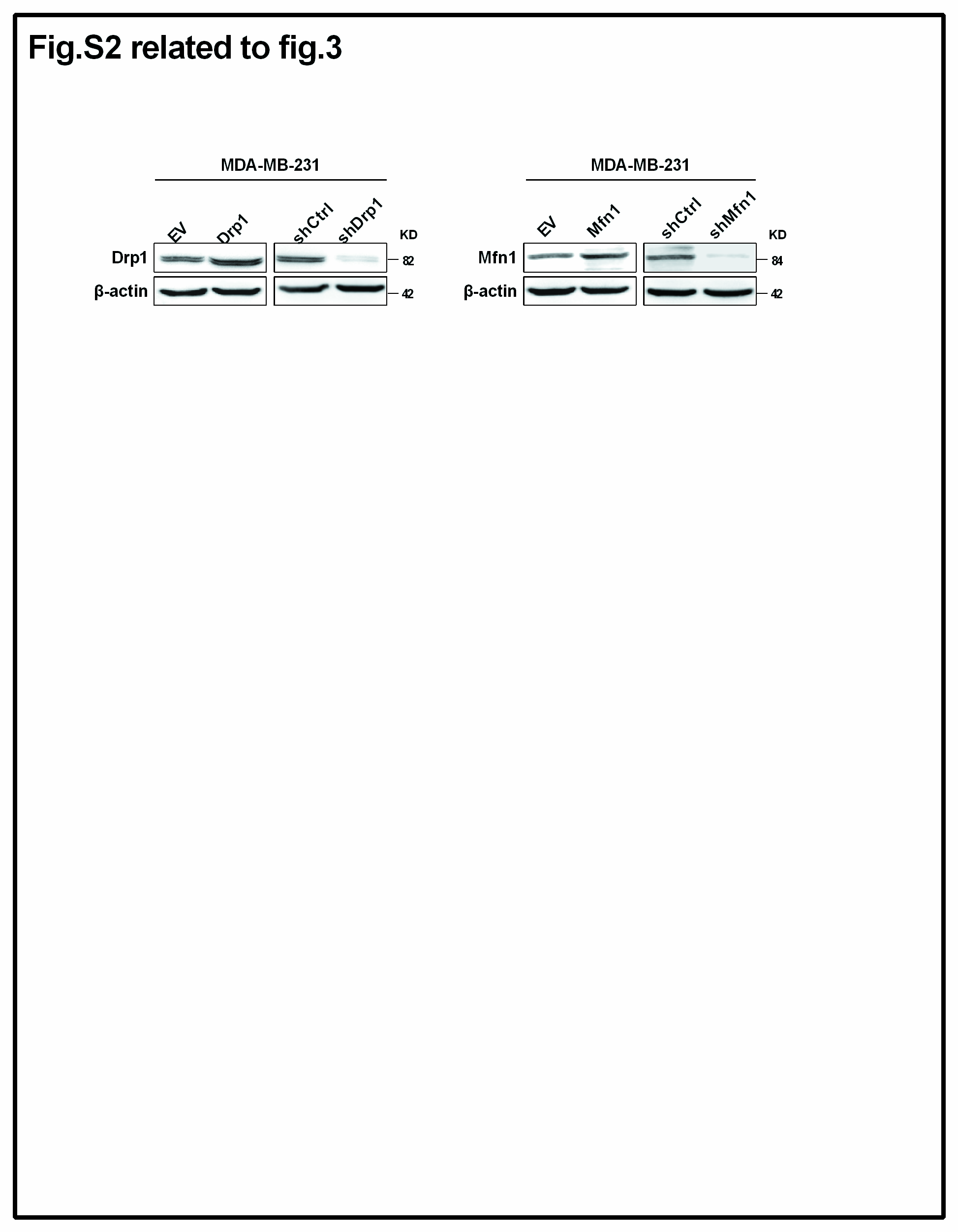

Supplement: Supplementary file 2 — Figure S2 related to fig 3 [file 41419_2018_1083_MOESM2_ESM.tif]

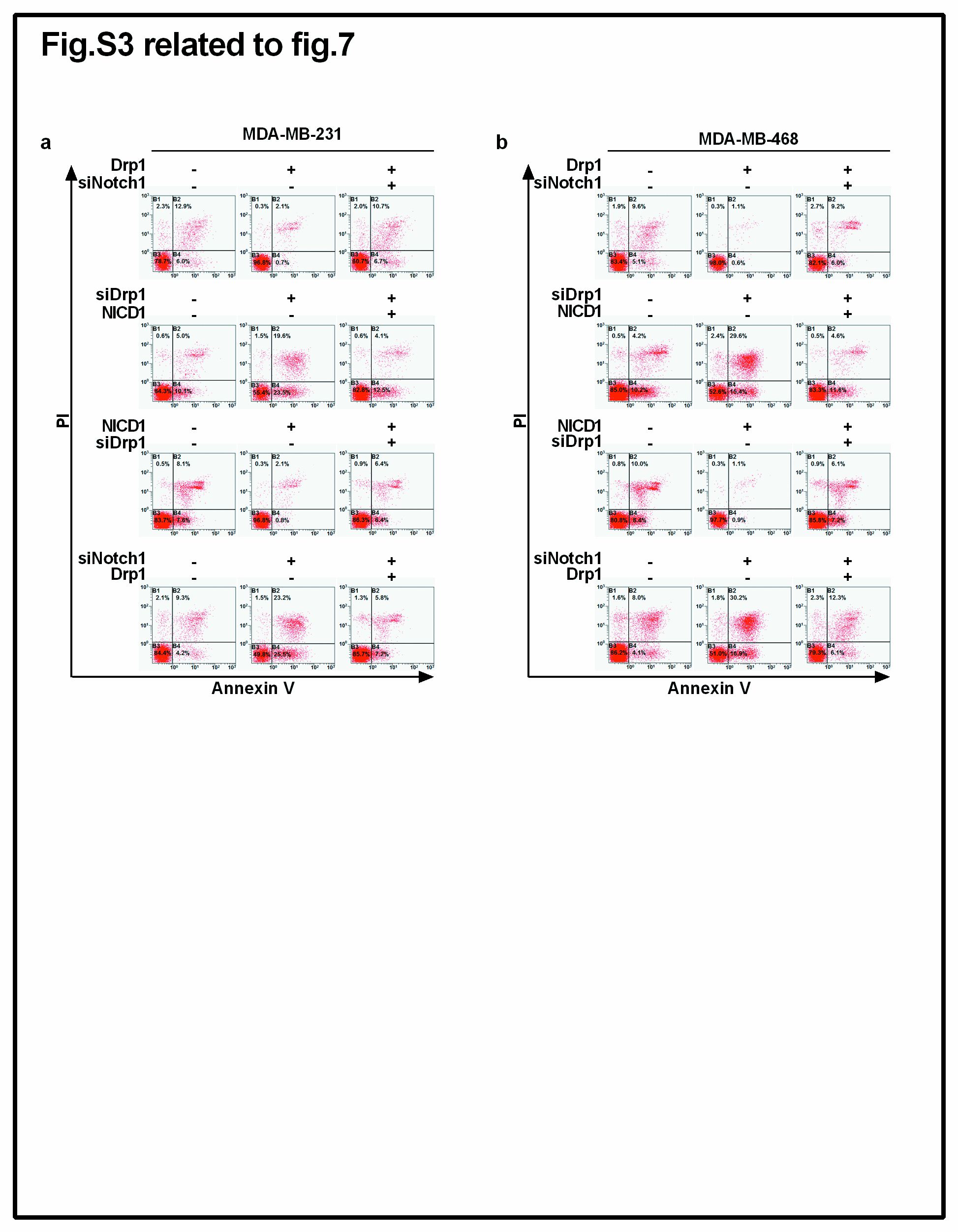

Supplement: Supplementary file 3 — Figure S3 related to fig 7 [file 41419_2018_1083_MOESM3_ESM.tif]

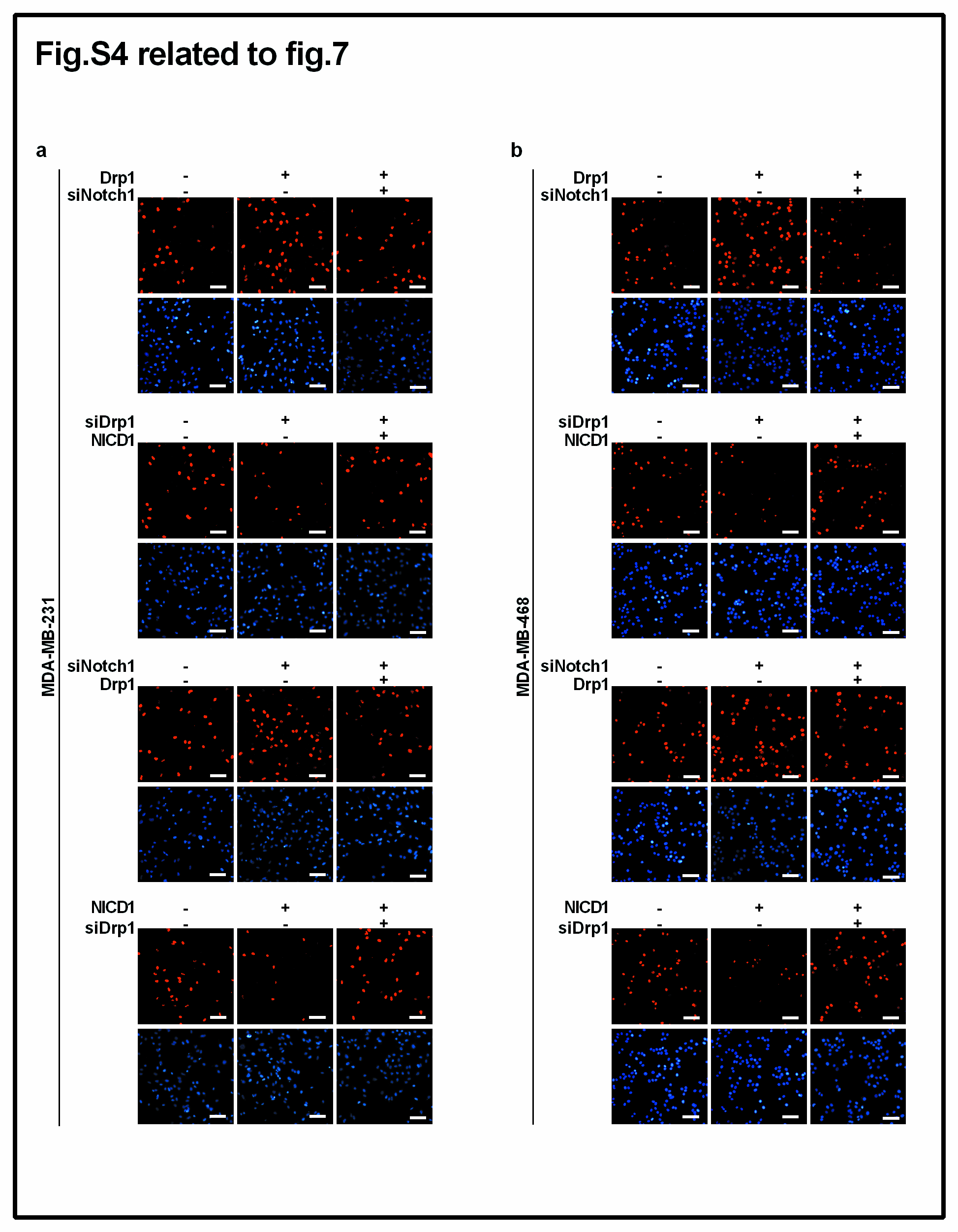

Supplement: Supplementary file 4 — Figure S4 related to fig 7 [file 41419_2018_1083_MOESM4_ESM.tif]
