# Supplementary material for: Positive feedback loop between mitochondrial fission and Notch signaling promotes survivin-mediated survival of TNBC cells
Source: Cell Death Dis. 2018 Oct 15;9(11):1050. doi: 10.1038/s41419-018-1083-y (PMC6189045; doi:10.1038/s41419-018-1083-y)
Supplement: Supplementary file 7 — Supplementary Information [file 41419_2018_1083_MOESM7_ESM.docx]

**Supplementary Information**

**Figure S1 related to Figure 2.** **The Drp1 and Mfn1 mRNA or protein level of TNCB cell after transfection for 48 h.** (a and b) qRT-PCR detected the mRNA levels of Drp1 and Mfn1 in the TNBC cells which were transfected with expression vectors or siRNAs as indicated, respectively. MDA-MB-231 and MDA-MB-468 were transfected with expression vectors or siRNA as indicated. The mRNA levels of Drp1 and Mfn1 were increased in cells overexpressed Drp1 or Mfn1 and were decreased in cells transfected with Drp1 or Mfn1 siRNA. Data was presented as mean ± SEM, n=3. *, *P*< 0.05; **, *P*< 0.01. (c and d) Western blot analysis to detect the protein levels of Drp1, p-Drp1 and Mfn1 in the TNBC cells which were transfected with expression vectors or siRNAs as indicated, respectively. Consistently, protein expression levels of Drp1 and p-Drp1 or Mfn1 were high in cells overexpressed Drp1 or Mfn1 and protein expression levels of Drp1 or Mfn1were low in cells transfected with Drp1 or Mfn1 siRNA. (e) Immunohistochemical staining analysis of p-Drp1 expression in TNBC and non-TNBC cells. *, *P*< 0.05; **, *P*< 0.01.

**Figure S2 related to Figure 3. The Drp1 and Mfn1 protein level of stably transfected MDA-MB-231 cells.** Western blot analysis to detect protein levels of Drp1 and Mfn1 in the stably transfected TNBC cells. 231 cells which stably transfected with Drp1 or Mfn1 had higher Drp1 or Mfn1 protein level than EV group. Cells which stably transfected with shDrp1 or shMfn1 had lower protein level than shCtrl group.

**Figure S3 related to Figure 7. Notch-mitochondrial fission positive feedback loop regulated cell** **apoptosis in TNBC cells.** Thapsigargin-induced apoptosis analyzed by flow cytometry in TNBC cells after transfection with different siRNAs or plasmids. (a) Drp1 overexpression inhibited apoptosis, which could be eliminated by Notch1 knockdown, while the activation of Notch signaling by NICD1 overexpression could rescue Drp1 silencing-induced apoptosis in both cell lines. (b) NICD1 overexpression reduced apoptosis of TNBC cells, which could be reversed by Drp1 knockdown, whereas Notch silencing-induced TNBC cell apoptosis could be rescued by Drp1 overexpression.

**Figure S4 related to Figure 7. Notch-mitochondrial fission positive feedback loop regulated cell proliferation in TNBC cells.** Cell proliferation determined by EdU analysis in TNBC cells 48 h after transfection with different expression vectors or siRNAs. (a) Notch1 silencing antagonized the proliferative effects of Drp1 overexpression, while NICD1 overexpression restored the proliferative capacity after Drp1 silencing. (b) Notch signaling-mediated cell proliferation also relied on Drp1 expression. The increased proliferative activity of TNBC cell with Drp1 or NICD1 overexpression can be significantly decreased by Notch1 or Drp1 knockdown.

**Figure S5 related to Figure 4. Mitochondrial fission inhibited TNBC cell apoptosis.** (a-d) Apoptosis of TNBC cells analyzed by flow cytometry 48h after siRNAs or plasmid transfection. Apoptosis was induced by 50 μM C2-ceramide for 24 h. *, *P*< 0.05; **, *P* < 0.01.

**Figure S6 related to Figure 8. Notch-mitochondrial fission positive feedback loop-induced survivin expression promoted TNBC cell survival.** (a and b) Changes of mitochondrial dynamics in TNBC cells after survivin overexpression. (c) Western blot analysis for Drp1 and survivin in TNBC cells with survivin overexpression. Scale bar: 5μm. *, *P*<0.05; **, *P*<0.01.

**Supplementary Table 1. Sequence of primers and siRNA.**

| Primers used in q-PCR analysis | |
| --- | --- |
| *Drp1* | 5’-ATGGAGGCGCTAATTCCTGTCATAA-3’ |
| *Mfn1* | 5’-ATGGCAGAACCTGTTTCTCCACTGA-3’ |
| *GAPDH* | 5’-GGAGCGAGATCCCTCCAAAAT-3’ |
| siRNA | |
| *Drp1* | 5’-ACUAUUGAAGGAACUGCAAAAUAUATT-3’ |
| *Mfn1* | 5’-GGAUCACAUUUUGUUGAAGTT-3’ |
| *Notch1* | 5’-GGGCUAACAAAGAUAUGCATT-3’ |
| *Survivin* | 5’-UAGCAAAAGGGACACUGCCTT-3’ |
| Control siRNA | 5’-UUCUCCGAACGUGUCACGUTT-3’ |
| Primers used in gene cloning | |
| *Drp1* | 5’-CCGGAATTCTAGCCAGTCTCCACATGAGC-3’ |
| *Mfn1* | 5’-ACGAATTCCTTGCCACCATGGCAGAACCT-3’ |
| *NICD1* | 5’-CTCGAGAATATGGTGCTGCTGTCCCGCAAG-3’ |

**Supplementary Table 2. Primary antibodies used for western blot andimmunohistochemistry.**

| Antibody | Company (Cat. No.) | Working dilutions |
| --- | --- | --- |
| Drp1 | abcam(ab56788) | WB:1/800 IHC:1/400 |
| p-Drp1^Ser637^ | abcam(ab193216) | WB:1/500 IHC:1/300 |
| Mfn1 | abcam(ab57602) | WB:1/500 IHC:1/200 |
| Notch1 | abcam(ab52627) | WB:1/800 IHC:1/400 |
| NICD1 | abcam(ab83253) | WB:1/700 |
| Survivin | Novus(NB500-201) | WB:1/800 |
| Ki67 | Fuzhou MXB BIOTEC CO. (RMA-0542) | IHC:1/150 |
